# Shedding of membrane complement inhibitors CD59 and CD46 into the circulation is associated with poor prognosis in acute coronary syndrome patients: a cohort study

**DOI:** 10.1186/s12967-024-05781-9

**Published:** 2024-11-10

**Authors:** Baojun Zhong, Ben King, Homa Waziri, Troels Yndigegn, Daniel Engelbertsen, Harry Björkbacka, Jan Nilsson, Isabel Goncalves, Anna M. Blom, Alexandru Schiopu

**Affiliations:** 1 Department of Translational Medicine, Lund University, Malmö, Sweden; 2 Department of Clinical Sciences Lund, Lund University, Lund, Sweden; 3 Department of Cardiology, Skåne University Hospital Lund, Lund, Sweden; 4 Department of Clinical Sciences Malmö, Lund University, Malmö, Sweden; 5 “N. Simionescu” Institute of Cellular Biology and Pathology, Bucharest, Romania; 6 Department of Internal Medicine, Skåne University Hospital, Lund, Sweden

**Keywords:** Acute coronary syndrome (ACS), Major adverse cardiovascular events (MACE), Heart failure (HF), Complement, CD46, CD59, Inflammation, Matrix metalloproteinases

## Abstract

**Introduction:**

The role of the complement inhibitory proteins CD46 and CD59 in the immune response to an acute coronary syndrome (ACS) is unknown. We investigated the relationships between the shedding of CD46 and CD59 into the circulation, reflected by plasma levels of soluble CD46 and CD59, and the risk for post-ACS complications.

**Methods:**

We measured plasma sCD46 and sCD59 in a cohort of 546 ACS patients within 24 h after hospital admission, and after 6-weeks in a subgroup of 114 patients. Study outcomes were incident heart failure (HF), major adverse cardiovascular events (MACE) and mortality during a median follow-up period of up to 3.3 years. Echocardiography at 1-year was performed in the follow-up subgroup.

**Results:**

Elevated sCD46 and sCD59 were correlated with increased levels of inflammatory mediators and metalloproteinases in plasma, and were associated with increased risk for MACE in Cox proportional hazard models adjusted for cardiovascular risk factors and revascularization [HR 95% CI 1.24 (1.02–1.52), p = 0.034 for sCD46 and 1.18 (1.00–1.38), p = 0.049 for sCD59]. Elevated sCD59 was also associated with higher incidence of HF [HR 95% CI 1.41 (1.15–1.74), p = 0.001], and with lower left ventricular ejection fraction at 1-year post-ACS (Spearman r = − 0.234, p = 0.020). We found no associations between plasma levels of the proteins at 6 weeks and outcomes.

**Conclusions:**

Shedding of the complement regulators CD46 and CD59 in plasma in the acute phase of ACS is associated with a negative prognosis. Plasma sCD46 and sCD59 could reflect the degree of local immune activation and serve as prognostic biomarkers in ACS patients.

**Supplementary Information:**

The online version contains supplementary material available at 10.1186/s12967-024-05781-9.

## Introduction

Acute coronary syndrome (ACS) and its complications are the leading causes of morbidity and mortality worldwide [1]. Acute coronary syndrome includes ST-elevation myocardial infarction (STEMI), non-ST-elevation myocardial infarction (NSTEMI), and unstable angina pectoris. The common complications post-ACS are heart failure (HF), recurrent coronary events and mortality. ACS is triggered by destabilization and rupture of atherosclerotic coronary plaques, and in the case of MI leads to myocardial damage, inflammation and loss of function. Excessive activation of complement pathways in the acute inflammatory phase of an ACS has been suggested to contribute to cardiomyocyte necrosis, apoptosis and myocardial injury. This hypothesis is supported by previous studies that found significantly higher levels of the soluble MAC components C5b-9 in patients who developed post-MI HF [2].

The complement system is an integral part of innate immunity, serving as the first line of defence against infections by enhancing the ability of phagocytic cells to identify and eliminate pathogens [3, 4]. Complement also plays an important role in the opsonization and phagocytosis of damaged cells and tissues. The proteins involved in the complement system circulate in an inactive form and are activated through the classical pathway (CP), the lectin pathway (LP) and the alternative pathway (AP). All pathways converge on the level of C3b activation followed by formation of the lytic membrane attack complex (MAC) [3].

Complement inhibitors are proteins that prevent excessive complement activation, safeguarding normal cells and tissues from damage. CD46, also known as membrane cofactor protein (MCP), is a member of the regulators of complement activation (RCA) gene cluster located on the long arm of chromosome 1 [5]. CD46 is present on all cell types except erythrocytes [5]. It acts as cofactor for serine protease factor I (FI), contributing to C3b degradation [6]. CD59 is a glycosylphosphatidylinositol (GPI)-anchored sarcolemmal complement inhibitor that plays a crucial role in inhibiting MAC formation. CD59 binds to C8 and C9, preventing the interaction of C9 with C5b678 and thereby preventing cell lysis or damage [7]. CD59 is widely expressed on the surface of various mammalian cells, including leukocytes, erythrocytes, platelets, epithelial cells, endothelial cells, and placental cells. The soluble form of CD59 (sCD59) has been detected in body fluids such as plasma, urine, tears, saliva, sweat and human milk [8, 9]. Under physiological conditions, the concentration of soluble CD59 in human plasma is low [10].

Increased expression of complement inhibitors occurs in response to complement activation, to prevent excessive tissue damage. MI patients have increased circulating concentration of sCD59 [9], and elevated mRNA expression of CD46 and CD59 in circulating leukocytes compared to healthy controls [11]. However, whether these complement inhibitors efficiently prevent cardiac injury and are associated with an improved post-ACS prognosis is unknown. In the current study, we measured plasma levels of sCD46 and sCD59 in a cohort of 546 ACS patients, at the time of the acute event and at 6-weeks follow-up, and examined their associations with cardiac function and remodelling at 1-year post-ACS and with the long-term risk to develop MACE, HF and mortality.

## Methods

### Study population

The study initially included 605 patients admitted to the Coronary Care Unit of Skåne University Hospital Malmö between October 2008 to December 2012 due to suspected ACS. We excluded 50 patients who did not meet the diagnostic criteria for ACS, and a further 9 patients due to missing samples. The final cohort consisted of 546 patients. Information on cardiovascular (CV) risk factors such as smoking, diabetes, hypertension, prevalent ACS and prevalent HF was obtained from the national Swedish Web-based system for Enhancement and Development of Evidence-based care in Heart disease Evaluated According to Recommended Therapies (SWEDEHEART). Kidney function was assessed using the estimated glomerular filtration rate (eGFR), calculated according to the Chronic Kidney Disease Epidemiology Collaboration (CKD-EPI) formula. All patients were required to sign an informed consent form before inclusion in the study. The study was approved by the Regional Ethics Committee for human studies in Lund, Sweden.

Venous blood samples were collected from all patients within 24 h of admission. To address knowledge gaps regarding post-ACS evolution and prognosis in the elderly, patients aged 75 years or older were included in a detailed follow-up subgroup consisting of 114 patients. In this subgroup, additional blood samples were collected 6-weeks after the index event and a follow-up echocardiography was performed at 1-year.

### Outcomes

The outcomes of interest were incident major adverse cardiovascular events (MACE) during follow-up, defined as recurrent ACS or CV death, incident HF, defined as hospitalization with a HF diagnosis, and total mortality. The median follow-up period was 2.28 years (IQR 1.21–3.60) for incident MACE, 2.10 years (IQR 1.12–3.42) for incident HF, and 3.30 years (IQR 2.33–4.52) for mortality. Incident events during follow-up were identified using data from the Swedish Hospital Discharge Register and the Swedish Cause of Death Register. All events were defined according to the International Classification of Diseases, 10th Revision (ICD–10) codes. Recurrent AMI was defined by codes I21 and I22, unstable angina by I20, and HF by I50.

### Biochemical analysis

Troponin T (TnT), creatinine and cystatin C for eGFR estimation were analysed at the Clinical chemistry unit of Laboratory Medicine Skåne. Plasma levels of sCD46 and sCD59 were analysed at the Science for Life Laboratory, Uppsala, Sweden by the Proximity Extension Assay technique, as previously described [12]. The same technique was used to measure plasma IL-6, IL-18, IL-8, CCL2, CCL3, CCL4, CX3CL1, MMP1, MMP3 and MMP7. The data for sCD46 and sCD59 are presented as arbitrary units (au).

### Echocardiography

As per the initial study protocol, patients aged 75 years or older were invited to a follow-up echocardiography examination at 1-year after the index hospitalization. A total of 96 patients successfully completed the following-up echocardiography. The left ventricular end-diastolic volume (LVEDV) and end-systolic volume (LVESV) were measured by the Simpson’s biplane method in the apical 4-chamber and 2-chamber views, averaged and indexed to body surface area (BSA). The left ventricular ejection fraction (LVEF) was calculated as the average LV stroke volume divided by the average LVEDV. Indexed LV mass (LVMi) was used as a measure of LV hypertrophy, and indexed left atrium volume (LAVi) was used to assess LA dilation. All echocardiography examinations were performed by experienced sonographers and the recordings were analysed using Xcelera (Philips) by a single examiner blinded to the clinical data.

### Statistical analysis

Differences in patient characteristics at baseline between the different outcome groups were examined by the Mann-Whitney U test for continuous variables and by the chi-square test for dichotomous variables. Kaplan–Meier survival analyses with log-rank tests and multivariate Cox proportional hazards analyses were used to assess the associations between biomarkers and outcomes. We used 3 different adjustment models: Model 1, unadjusted; Model 2 adjusted for age and sex; and Model 3 adjusted for age, sex and the established prognostic factors diabetes mellitus, hypertension, smoking, prevalent ACS and prevalent HF before the index event, as well as coronary revascularization during the index hospitalization. Before analysis, skewed variables were logarithmically transformed. The correlations between plasma biomarkers, cytokines, chemokines, MMPs and echocardiographic parameters were analysed by the Spearman’s rank test. The associations were considered significant at p < 0.05. All the calculations were performed by SPSS 29.0.0 (IBM software, Armonk NY). The Kaplan–Meier analysis was performed by R, version 4.3.1.

## Results

### Baseline characteristics of the cohort

The baseline characteristics of the study population, stratified by incident MACE, HF and mortality during follow-up, are shown in Table 1 and Supplementary tables 1 and 2. The patients with a poor prognosis were older and had a higher prevalence of hypertension, diabetes, previous ACS, previous HF and lower eGFR at baseline. Additionally, a higher percentage of the patients re-admitted with a HF diagnosis had diabetes at baseline, and smoking was more prevalent in patients that died during follow-up. The baseline levels of sCD46 and sCD59 in plasma were significantly elevated in the patients with a poor prognosis, regardless of the outcome.

**Table 1:** Difference in baseline characteristics between patients with and without heart failure (HF) during follow-up

Characteristics	All patients	Incident HF	No incident HF	P
	N = 546	N = 41	N = 505	
Age (years)	67 (59–77)	77 (71.5–84.5)	66 (58–76)	< 0.001
Male gender, n (%)	398 (72.9)	28 (68.3)	370 (73.3)	0.491
Hypertension, n (%)	294 (53.8)	34 (82.9)	260 (51.5)	< 0.001
Smoking, n (%)	137 (25.1)	10 (24.4)	127 (25.1)	0.914
Diabetes, n (%)	131 (24.0)	20 (48.8)	111 (22.0)	< 0.001
BMI (kg/m^2^)	26.9 (24.3–29.8)	27.2 (23.9–29.8)	26.9 (24.3–29.8)	0.608
eGFR (mL/min/1.73 m^2^)	72.0 (53.1–94.6)	46.0 (31.6–64.2)	73.9 (55.8–96.3)	< 0.001
Index cardiac event, n (%)				0.340
STEMI	189 (34.6)	11 (26.8)	178 (35.2)	
NSTEMI	306 (56.0)	24 (58.5)	282 (55.8)	
Unstable angina	51 (9.3)	6 (14.6)	45 (8.9)	
Prevalent event, n (%)				
ACS	156 (28.6)	19 (46.3)	137 (27.1)	0.009
HF	56 (10.3)	15 (36.6)	41 (8.1)	< 0.001
Revascularization	379	23	356	0.054
PCI	323	19	304	0.083
CABG	56	4	52	0.913
CD46 (au)	2.10 (1.06–3.17)	4.18 (4.01–4.64)	3.98 (3.75–4.27)	< 0.001
CD59 (au)	0.56 (0.34–0.83)	1.00 (0.73–1.55)	0.54 (0.33–0.77)	< 0.001
Discharge medication (%)				
ACE inhibitor	401 (73.4)	31 (75.6)	370 (73.3)	0.759
ARB	86 (15.8)	5 (12.2)	81 (16.0)	0.513
Warfarin	27 (4.9)	6(14.6)	21 (4.2)	0.003
ASA	525 (96.2)	39 (95.1)	486 (96.2)	0.669
P2Y12 inhibitor	460 (84.2)	32 (78.0)	428 (84.8)	0.244
Beta-blocker	501(91.8)	39 (95.1)	462 (91.5)	0.435
Statin	525 (96.2)	35 (85.4)	490 (97.0)	< 0.001

ACS: acute coronary syndrome; BMI: body mass index; eGFR: estimated glomerular filtration rate; NSTEMI: non-ST elevation myocardial infarction; STEMI: ST elevation myocardial infarction; ACE inhibitor: angiotensin-converting enzyme inhibitor; ARB: angiotensin II receptor blocker; ASA: acetylsalicylic acid

### Elevated acute-phase sCD46 and sCD59 are associated with systemic inflammation, tissue damage and poor post-ACS prognosis

We examined the correlations between plasma levels of sCD46 and sCD59, infarction size, systemic inflammation, and the intensity of immune activation and tissue turnover during the acute phase of the ACS. We found no correlations with Troponin T (TnT) and weak significant correlations with high-sensitivity C-reactive protein (hsCRP) in plasma (Table 2). Plasma sCD46 and sCD59 were also weakly correlated with the concentration of the inflammatory cytokines IL-6 and IL-18, and presented moderate correlations with IL-8, CCL2, CCL3, CCL4, and CX3CL1, chemokines involved in innate immune cell recruitment. Importantly, we also found moderate significant correlations with plasma levels of matrix metalloproteinases 1, 3, and 7 (MMP1, MMP3, MMP7), potentially reflecting processes involved in cardiac tissue damage and turnover during ACS (Table 2).

**Table 2:** Correlations between sCD46 and sCD59 in plasma and markers of immune activation and tissue damage

		CD46		CD59	
		r	p	r	p
Infarction size Systemic inflammation	TnT	− 0.038	0.385	0.022	0.619
	hsCRP	0.085	0.052	0.154	< 0.001
Inflammatory cytokines	IL-6	0.105	0.017	0.205	< 0.001
	IL-18	0.158	< 0.001	0.157	< 0.001
Chemokines	IL-8	0.265	< 0.001	0.292	< 0.001
	CCL2	0.298	< 0.001	0.276	< 0.001
	CCL3	0.325	< 0.001	0.355	< 0.001
	CCL4	0.239	< 0.001	0.236	< 0.001
	CX3CL1	0.293	< 0.001	0.301	< 0.001
MMPs	MMP1	0.246	< 0.001	0.174	< 0.001
	MMP3	0.178	< 0.001	0.235	< 0.001
	MMP7	0.314	< 0.001	0.379	< 0.001

TnT: Troponin T, hsCRP: high-sensitivity C-reactive protein, MMPs: Matrix metalloproteinases

**Fig. 1:**
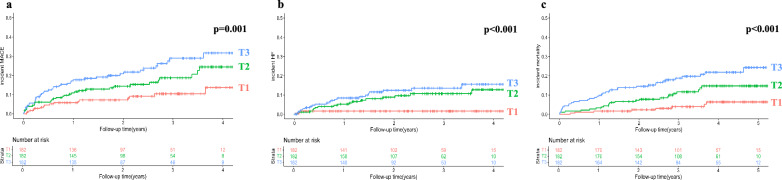
Plasma sCD46 and post-ACS prognosis. Kaplan–Meier 1-minus survival curves showing incident MACE (**a**), HF (**b**) and mortality (**c**) post-MI by plasma sCD46 tertiles at baseline. The p-values refer to the difference in incident outcomes across tertiles, calculated with the log-rank test

**Fig. 2:**
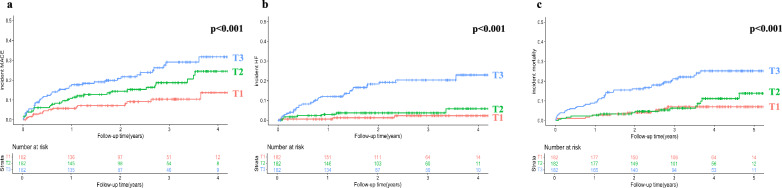
Plasma sCD59 and post-ACS prognosis. Kaplan–Meier 1-minus survival curves showing incident MACE (**A**), HF (**B**) and mortality (**C**) post-AMI by plasma sCD59 tertiles at baseline. The p-values refer to the difference in incident outcomes across tertiles, calculated with a log-rank test

We used unadjusted Kaplan–Meier survival analyses with log-rank tests to examine the incidence of MACE, HF, and mortality by baseline sCD46 and sCD59 tertiles. We found significantly increased incidence of MACE and mortality with increasing sCD46 tertile, and patients within the top two sCD46 tertiles had a higher incidence of HF compared to the bottom tertile (Fig. 1). Patients with the highest levels of sCD59 had significantly higher incidence of all considered outcomes compared to the lower tertiles, while there was no difference between tertiles 1 and 2 (Fig. 2). Patients with plasma sCD59 in tertile 1 and 2 had a strikingly low incidence of incident HF, with 3 events in tertile 1 and 7 events in tertile 2, compared to 31 events among patients in the highest tertile of baseline sCD59.

Further, we investigated the associations between sCD46 and sCD59 in plasma at the time of the ACS and the considered outcomes in Cox proportional regression analyses sequentially adjusted for age, sex and potential confounders of prognostic significance. sCD46 was significantly associated with incident MACE, independently of age, sex, CV risk factors, prevalent ACS, prevalent HF and revascularization (Table 3, Models 1–3). sCD46 was also associated with incident HF and mortality, but the associations lost significance after adjustment for potential confounders (Table 3, Model 3). In contrast, sCD59 was independently associated with both MACE and HF in all regression models (Table 3, Models 1–3). Additionally, the association between baseline sCD59 and HF remained significant after further adjustment for kidney function, measured as eGFR at baseline (HR = 1.29, 95% CI 1.01–1.63, p = 0.040). The association between sCD59 and total mortality was lost in the fully adjusted model (Table 3, Model 3). We also performed a subgroup analysis including only the 378 patients in our cohort that were younger than 75 years of age at baseline. The associations remained significant in this subgroup, demonstrating the robustness of the results (Supplementary Table 3). The strongest association was between CD59 and incident HF, followed by the association between CD46 and MACE. The relationship between CD59 and MACE maintained borderline significance after full adjustment for CV risk factors and revascularization (Supplementary Table 3).

**Table 3:** Associations between CD46 and CD59 at baseline and outcomes

Biomarkers	MACE (N = 90)			HF (N = 41)			Total mortality (N = 66)		
	HR*	CI	P	HR*	CI	P	HR*	CI	P
CD46									
Model 1	1.42	1.20–1.68	< 0.001	1.59	1.27–1.99	< 0.001	1.41	1.16–1.71	< 0.001
Model 2	1.32	1.09–1.59	0.005	1.47	1.14–1.90	0.003	1.18	0.94–1.48	0.143
Model 3	1.24	1.02–1.52	0.034	1.26	0.96–1.66	0.097	1.07	0.84–1.36	0.578
CD59									
Model 1	1.45	1.28–1.64	< 0.001	1.69	1.46–1.95	< 0.001	1.51	1.32–1.74	< 0.001
Model 2	1.27	1.10–1.47	0.001	1.51	1.28–1.78	< 0.001	1.21	1.02–1.44	0.032
Model 3	1.18	1.00–1.38	0.049	1.41	1.15–1.74	0.001	1.07	0.88–1.31	0.490

^*^HR is expressed per 1-SD increase in baseline CD46 and CD59 values

Adjustment models:

Model 1: unadjusted

Model 2: age and sex

Model 3: age, sex, CV risk factors (diabetes mellitus, hypertension, smoking), prevalent ACS, prevalent HF, revascularization (PCI / CABG)

ACS: acute coronary syndrome; MACE: major adverse cardiovascular event; HF: heart failure; HR: hazard ratio; CI: confidence interval

In order to assess whether the relationships extend beyond the acute phase, we evaluated the associations between plasma biomarkers measured at the 6-weeks follow-up time point and the considered outcomes, using the same adjustment models (Table 4). We found no significant associations between sCD46 and sCD59 and the outcomes at this time point, regardless of the adjustment model, suggesting that the activation of the complement pathways is primarily relevant during the acute phase of the ACS and the complement inhibitors have no predictive value during follow-up.

**Table 4:** Associations between CD46 and CD59 at 6 weeks and outcomes

Model	MACE (N = 30)			HF (N = 14)			Total mortality (N = 20)		
	HR	CI	P	HR	CI	P	HR	CI	P
CD46									
Model 1	0.88	0.58–1.34	0.547	0.82	0.44–1.52	0.525	0.99	0.61–1.60	0.955
Model 2	0.78	0.50–1.20	0.251	0.79	0.42–1.47	0.454	0.90	0.55–1.45	0.655
Model 3	0.79	0.50–1.26	0.326	0.69	0.34–1.40	0.303	0.79	0.44–1.40	0.418
CD59									
Model 1	1.24	0.93–1.64	0.143	0.94	0.52–1.68	0.828	1.22	0.83–1.80	0.311
Model 2	1.10	0.80–1.52	0.548	0.86	0.45–1.61	0.629	1.02	0.64–1.62	0.944
Model 3	1.07	0.78–1.46	0.667	0.80	0.40–1.60	0.529	0.93	0.54–1.58	0.778

Adjustment models:

Model 1: unadjusted

Model 2: age and sex

Model 3: age, sex, CV risk factors (diabetes mellitus, hypertension, smoking), prevalent ACS, prevalent HF, revascularization (PCI/CABG)

ACS: acute coronary syndrome; MACE: major adverse cardiovascular event; HF: heart failure; HR: hazard ratio; CI: confidence interval

### High plasma CD59 is associated with increased LV dysfunction and remodeling at 1-year after the acute coronary event

To further investigate the cardiac substrate of the relationships between sCD46 and sCD59 with incident HF during follow-up, we investigated the correlations between the soluble proteins and echocardiographic parameters of cardiac structure and function at 1-year post-ACS. sCD46 was positively correlated with LVMi but did not present any other correlations with LV volumes or function (Table 5). In contrast, sCD59 presented a stronger correlation with LVMi and was also positively correlated with LV volumes, suggestive of accelerated LV remodeling in patients with high sCD59 at baseline. Importantly, we also found a significant negative correlation between sCD59 and the LV systolic function during follow-up (Table 5), supporting the observed association with incident HF.

**Table 5:** Correlations between plasma sCD46 and sCD59, cardiac structure and function 1-year post-ACS

	CD46		CD46 at 6 weeks		CD59		CD59 at 6 weeks	
	r	p	r	p	r	p	r	p
LVMi	0.269	0.005	0.107	0.288	0.400	< 0.001	0.227	0.023
LVEDVi	0.183	0.074	0.209	0.048	0.185	0.072	0.076	0.474
LVESVi	0.162	0.114	0.200	0.059	0.213	0.038	0.062	0.559
LVEF	− 0.107	0.292	− 0.122	0.245	− 0.234	0.020	− 0.104	0.325
LAVi	0.069	0.487	0.047	0.648	0.127	0.196	0.180	0.075

LVMi: Left ventricular mass indexed to body surface area (BSA); LVEDVi: Left ventricular end-diastolic volume indexed; LVESVi: Left ventricular end-systolic volume indexed; LVEF, Left ventricular ejection fraction; LAVi: Left atrial volume indexed

r: Spearman correlation

## Discussion

In this prospective cohort study, we explored the associations between plasma levels of the soluble form of the complement inhibitors CD46 and CD59 and long-term prognosis after an acute coronary event. After adjusting for age, sex and potential confounders, we found that elevated levels of both proteins were independently associated with increased risk for recurrent coronary events, and sCD59 was strongly associated with the risk for HF hospitalization. The link between sCD59 and incident HF was further supported by positive correlations with LV mass and volumes, indicative of accelerated remodelling, and a negative association with LV systolic function at 1-year after the index event.

During the acute phase of ACS, danger-associated molecular patterns (DAMPs) released by cell necrosis bind to pattern recognition receptors (PRRs) on immune cells, triggering inflammatory responses [13]. PRRs such as mannose-binding lectin (MBL), and collectins trigger the complement system through the lectin pathway [14]. The activated complement fragments trigger an inflammatory response [6] and excessive complement activation was found to exacerbate cardiomyocyte injury [15]. Previous studies have shown that MBL levels are elevated during cardiac ischemia/reperfusion and contribute to cardiomyocyte damage [16, 17]. Accordingly, Weisman et al. and Banz et al. reported that blocking complement activation by soluble CR1 in animal models of MI led to smaller injuries and improved function [18, 19]. These results suggest that the complement system plays an important role in cardiac injury post-MI, and that inhibition of complement pathways might be a useful treatment to prevent post-MI complications.

CD46 and CD59 inhibit excessive complement activation, and higher deposition of these inhibitors in the ischemic myocardial tissue might confer protection against development of post-ACS complications. Early work by Väkevä et al. suggested that the loss of CD59 from the ischemic myocardium allows MAC formation, leading to increased tissue damage [20]. Using biopsies from human infarcted myocardium collected between 1 and 14 days post-mortem, the authors found lower expression or absence of CD59 in the infarcted areas, with concomitant MAC deposition in CD59-negative regions. CD59 was present in small vesicles between the infarcted area and normal tissues, suggesting that it might have been lost through shedding [20]. These findings have been confirmed in a rat model of myocardial ischemia, demonstrating increased presence of complement factors in the myocardium within hours after the acute event. CD59 expression was lost from day 1 onwards and was associated with concomitant MAC deposition [21]. The role of MAC deposition in myocardial loss and dysfunction is further supported by studies showing MAC expression in biopsies from failing human hearts, but not in healthy myocardium [22].

Following its shedding from the myocardium, CD59 can be measured in plasma as sCD59. Plasma levels of sCD59 were previously found to be significantly increased at 4 h and 24 h post-MI compared to healthy controls. Importantly, there was a strong correlation between plasma sCD59 and circulating levels of soluble components of the terminal complement pathway C5b-9, suggesting that sCD59 shedding could allow MAC deposition in the post-ischemic myocardium [9]. There are no previous studies investigating the possible associations between plasma sCD59 and prognosis in ACS patients. However, indications that associations between sCD59 and the extent of tissue damage might exist have been provided by an earlier study on 68 patients with recovery of spontaneous circulation after cardiac arrest [23]. Serum levels of sCD59, sC5b-9, C5a, C3a, C3b, C1q, MBL, Bb, TNF-α, IL-6, neuron-specific enolase (NSE) and S100β have been found to be elevated in these patients compared to controls. sCD59 was positively correlated with sC5b-9, TNF-α, IL-6, S100β and NSE, suggesting that CD59 shedding is proportional with the degree of MAC activation and tissue inflammation. Plasma sCD59 on days 1, 3 and 7 post-cardiac arrest was the strongest predictor of a poor neurological prognosis and mortality at 28 days, supporting its value as a prognostic biomarker [23].

CD46 acts as a cofactor for FI to induce the degradation of C3b and C4b [5]. So far, there are very few studies focusing on the expression and role of CD46 during and after AMI. Yan et al*.* have found that genes encoding complement proteins, receptors and inhibitors, including CD46 and CD59, were overexpressed in circulating peripheral blood mononuclear cells from AMI patients compared to healthy controls and patients with stable angina pectoris. These findings suggest activation of the complement system and an increase in its inhibitors on circulating immune cells during AMI [24]. A necropsy study on myocardial specimens collected from 50 deceased patients within 2 days post-MI has shown elevated immunoreactivity for both CD46 and CD59 compared to myocardium of controls who had died due to accidents. The expression of complement proteins, MAC and the complement inhibitors was predominantly located in the coronary endothelium, suggesting deposition of complement factors from the blood stream, but was also present on single cells infiltrating the necrotic myocardium, possibly immune cells [25].

We also found that plasma levels of sCD46 and sCD59 were significantly correlated with the levels of inflammatory cytokines, chemokines and metalloproteinases. To our knowledge, this is the first study to demonstrate positive associations between plasma levels of soluble complement regulatory proteins and a broad spectrum of pro-inflammatory immune mediators in the acute post-ACS period. We hypothesize that both CD46 and CD59 shedding and the potent cytokine and chemokine release into the circulation are simultaneously involved in the acute phase of the post-ischemic immune response and myocardial inflammation. However, our study design cannot directly address this hypothesis, as we cannot establish causal or temporal links between these events. Elevated plasma IL-6 has previously been shown to be associated with poor prognosis post-MI [26]. High IL-8 has also been associated with larger infarction, worse LV recovery and adverse prognosis in STEMI patients [27]. CCL2, CCL3, CCL4 and CX3CL1 are important chemokines involved in inflammatory and reparatory immune processes [28]. Increased levels of CCL3 and CX3CL1 in plasma have previously been linked with increased risk for future MACE in ACS patients, suggesting that the immune responses driven by these chemokines in the acute phase are predominantly detrimental [29, 30]. Matrix metalloproteinases (MMPs) are enzymes involved in tissue damage, repair and remodelling through digestion of extracellular matrix proteins. High plasma MMP3 in the acute phase has been linked to LV dysfunction and increased mortality post-MI. Evidence for a potential direct detrimental role of MMP7 in MI has been provided by experimental work showing that elevated levels of the metalloproteinase post-MI affect electrical conductivity in the myocardium, leading to increased animal mortality. Deletion of MMP7 reversed these effects [31].

### Study limitations

Our study has some limitations that have to be acknowledged. As this is an association study, it cannot provide causal links between sCD59, sCD46 and adverse events in this population. As discussed above, we speculate that the shedding of CD46 and CD59 from the myocardium and their subsequent increase in the circulation may lead to impaired tissue protection, leading to MAC-mediated cellular damage. Secondly, follow-up blood samples and echocardiographic data were only collected from a small sub-group of patients over 75 years of age. Consequently, we cannot exclude that this sub-group might have been underpowered to detect associations between sCD46 and sCD59 at 6-weeks and the outcomes, and the described correlations between sCD59 and echocardiographical parameters of cardiac dysfunction and remodelling cannot be directly extrapolated to younger subjects. However, the associations between sCD59 and incident HF during follow-up were valid in the entire study population. Lastly, as the data is expressed as arbitrary units, we cannot establish cut-off values for the use of sCD46 and sCD59 as a prognostic biomarker for MACE and HF in clinical practice. Our study should be considered as hypothesis-generating.Further studies based on measurements of absolute values by other methods are necessary to confirm our findings and define the potential use of sCD46 and sCD59 as biomarkers in clinical practice.

### Conclusion

In conclusion, we show that high sCD59 levels in plasma during the acute phase of an ACS are associated with long-term risk for recurrent cardiovascular events, cardiac remodelling and dysfunction, and incident HF. sCD46 also presented associations with incident MACE, but not with HF. We speculate that sCD46 and sCD59 release from the infarcted myocardium might allow MAC deposition and complement-induced cardiac injury, reflected by elevated levels of immune mediators and metalloproteinases. Our data suggest that therapeutic restoration of these complement inhibitory mechanisms or disruption of MAC deposition in the ischemic myocardium might reduce damage and prevent adverse events during follow-up. The value of sCD46 and sCD59 as independent prognostic biomarkers or as a surrogate biomarkers to monitor the efficiency of therapeutic interventions targeting immune and inflammatory mechanisms has to be confirmed in future studies.

## Supplementary Information

Additional file 1

## Data Availability

The datasets used and/or analysed during the current study are available from the corresponding author on reasonable request.

## Abbreviations

ACEi: Angiotensin-converting enzyme inhibitor
ACS: Acute coronary syndrome
AMI: Acute myocardial infarction
AP: Alternative pathway
ARB: Angiotensin II receptor blocker
ASA: Acetylsalicylic acid
au: Arbitrary units
BMI: Body mass index
BSA: Body surface area
CABG: Coronary artery bypass graft surgery
CI: Confidence interval
CP: Classical pathway
CPB: Cardiopulmonary bypass
CV: Cardiovascular
DAMPs: Danger-associated molecular patterns
ECM: Extracellular matrix
eGFR: Estimated glomerular filtration rate
FI: Factor I
GPI: Glycosylphosphatidylinositol
HF: Heart failure
HR: Hazard ratio
hsCRP: high-sensitivity C-reactive protein
IQR: Interquartile range
ICD: International Classification of Diseases
LAVi: Left atrial volume indexed
LP: Lectin pathway
LVMi: Left ventricular mass indexed to body surface area (BSA)
LVEDVi: Left ventricular end-diastolic volume indexed to body surface area (BSA)
LVEF: Left ventricular ejection fraction
LVESVi: Left ventricular end-systolic volume indexed to body surface area (BSA)
MAC: Membrane attack complex
MACE: Major adverse cardiovascular events
MBL: Mannose-binding lectin
MCP: Membrane cofactor protein
MI: Myocardial infarction
MMPs: Matrix metalloproteinases
NSE: Neuron-specific enolase
NSTEMI: Non-ST-elevation myocardial infarction
PCI: Percutaneous coronary intervention
PRRs: Pattern recognition receptors
RCA: Regulators of complement activation
sCD46: Soluble form of CD46
sCD59: Soluble form of CD59
STEMI: ST-elevation myocardial infarction
SWEDEHEART: System for Enhancement and Development of Evidence-based care in Heart disease Evaluated According to Recommended Therapies
TnT: Troponin T
