# Supplementary material for: Shedding of membrane complement inhibitors CD59 and CD46 into the circulation is associated with poor prognosis in acute coronary syndrome patients: a cohort study
Source: J Transl Med. 2024 Nov 10;22:1011. doi: 10.1186/s12967-024-05781-9 (PMC11550518; doi:10.1186/s12967-024-05781-9)
Supplement: Supplementary file 1 — Additional file 1 [file 12967_2024_5781_MOESM1_ESM.docx]

**Shedding of membrane complement inhibitors CD59 and CD46 into the circulation is associated with poor prognosis in acute coronary syndrome patients: a cohort study**

Baojun Zhong^1^, Ben King^1^, Homa Waziri^1^, Troels Yndigegn^2, 3^, Daniel Engelbertsen^4^, Harry Björkbacka^4^, Jan Nilsson^4^, Isabel Goncalves^4^, Anna M. Blom^1^, Alexandru Schiopu^1,5,6^

Affiliations

^1^ Department of Translational Medicine, Lund University, Malmö, Sweden

^2^ Department of Clinical Sciences Lund, Lund University, Lund, Sweden

^3^ Department of Cardiology, Skåne University Hospital Lund, Lund, Sweden

^4^ Department of Clinical Sciences Malmö, Lund University, Malmö, Sweden

^5^ “N. Simionescu” Institute of Cellular Biology and Pathology, Bucharest, Romania

^6^ Department of Internal Medicine, Skåne University Hospital, Lund, Sweden

**Supplementary Material**

**Supplementary Table 1.** Difference in baseline characteristics between patients with and without incident MACE during follow-up.

| **Characteristics** | **All patients** | **Patients with incident MACE** | **Patients without incident MACE** | **P** |
| --- | --- | --- | --- | --- |
|  | **N=546** | **N=90** | **N=456** |  |
| Age (years) | 67 (59–77) | 77 (65.5–85) | 66 (58–75) | <0.001 |
| Male gender, n (%) | 398 (72.9) | 64 (71.1) | 334 (73.2) | 0.677 |
| Hypertension, n (%) | 294 (53.8) | 58 (64.4) | 236 (51.8) | 0.027 |
| Smoking, n (%) | 137 (25.1) | 19 (21.1) | 118 (25.9) | 0.341 |
| Diabetes, n (%) | 131 (24.0) | 25(27.8) | 106 (23.2) | 0.358 |
| BMI (kg/m^2^) | 26.9 (24.3–29.8) | 26.1 (23.7–29.4) | 27.1 (24.4–29.8) | 0.187 |
| eGFR (mL/min/1.73m^2^) | 72.0 (53.1–94.6) | 54.9 (34.5–75.8) | 74.7 (57.2–96.5) | <0.001 |
| Index cardiac event, n (%) |  |  |  | 0.138 |
| STEMI | 189 (34.6) | 29 (32.2) | 160 (35.1) |  |
| NSTEMI | 306 (56.0) | 57 (63.3) | 249 (54.6) |  |
| Unstable angina | 51 (9.3) | 4 (4.4) | 47 (10.3) |  |
| Prevalent event, n (%) |  |  |  |  |
| ACS | 156(28.6) | 40(44.4) | 116 (25.4) | <0.001 |
| HF | 56 (10.3) | 19 (21.1) | 37 (8.1) | <0.001 |
| CD46 (au) | 2.10 (1.06-3.17) | 4.15 (3.90–4.60) | 3.96 (3.74-4.23) | <0.001 |
| CD59 (au) | 0.56 (0.34-0.83) | 0.77 (0.47-1.27) | 0.54 (0.32-0.75) | <0.001 |
| Discharge medication (%) |  |  |  |  |
| ACE inhibitor | 401 (73.4) | 52(57.8) | 349 (76.5) | <0.001 |
| ARB | 86 (15.8) | 20 (22.2) | 66 (14.5) | 0.058 |
| Warfarin | 27 (4.9) | 6(6.7) | 21 (4.6) | 0.396 |
| ASA | 525 (96.2) | 84 (93.3) | 441 (96.7) | 0.285 |
| P2Y12 inhibitor | 460 (84.2) | 80 (88.9) | 379 (83.3) | 0.119 |
| Betablocker | 501(91.8) | 75 (83.3) | 426 (93.4) | 0.004 |
| Statin | 525 (96.2) | 76 (84.4) | 449 (98.5) | <0.001 |

ACS: acute coronary syndrome; BMI: body mass index; eGFR: estimated glomerular filtration rate; TNT: Troponin T; MACE: major adverse cardiovascular event; NSTEMI: non-ST elevation myocardial infarction; STEMI: ST elevation myocardial infarction; ACE inhibitors: angiotensin-converting enzyme inhibitor; ARB: angiotensin II receptor blocker; ASA: acetylsalicylic acid.

**Supplementary Table 2.** Difference in baseline characteristics between deceased patients and alive patients during follow-up.

| **Characteristics** | **All patients** | **Dead** | **Alive** | **P** |
| --- | --- | --- | --- | --- |
|  | **N=546** | **N=66** | **N=480** |  |
| Age (years) | 67 (59–77) | 81.5 (74.8–86.0) | 66 (58–74) | <0.001 |
| Male gender, n (%) | 398 (72.9) | 47 (71.2) | 351 (73.1) | 0.743 |
| Hypertension, n (%) | 294 (53.8) | 50 (75.8) | 244 (50.8) | <0.001 |
| Smoking, n (%) | 137 (25.1) | 10 (15.2) | 127 (26.5) | 0.047 |
| Diabetes, n (%) | 131 (24.0) | 22 (33.3) | 109 (22.7) | 0.058 |
| BMI (kg/m^2^) | 26.9 (24.3–29.8) | 25.9 (23.4–29.8) | 27.1 (24.4–29.8) | 0.173 |
| eGFR (mL/min/1.73m^2^) | 72.0 (53.1–94.6) | 49.8 (34.2–68.9) | 74.0 (57.0–96.5) | <0.001 |
| Index cardiac event, n (%) |  |  |  | 0.102 |
| STEMI | 189 (34.6) | 16 (24.2) | 173 (36.0) |  |
| NSTEMI | 306 (56.0) | 45 (68.2) | 261 (54.4) |  |
| Unstable angina | 51 (9.3) | 5 (7 .6) | 46 (9.6) |  |
| Prevalent event, n (%) |  |  |  |  |
| ACS | 156 (28.6) | 33 (50.0) | 123 (25.6) | <0.001 |
| HF | 56 (10.3) | 18 (27.3) | 38 (7.9) | <0.001 |
| CD46 (au) | 2.10 (1.06-3.17) | 4.20 (3.97-4.52) | 3.97 (3.74-4.25) | <0.001 |
| CD59 (au) | 0.56 (0.34-0.83) | 0.89 (0.47-1.28) | 0.54 (0.33-0.75) | <0.001 |
| Discharge medication (%) |  |  |  |  |
| ACE inhibitor | 401 (73.4) | 36 (54.5) | 365 (76.0) | <0.001 |
| ARB | 86 (15.8) | 14 (21.2) | 72 (15.0) | 0.175 |
| Warfarin | 27 (4.9) | 7 (10.6) | 20 (4.2) | 0.021 |
| ASA | 525 (96.2) | 59 (89.4) | 466 (97.1) | 0.011 |
| P2Y12 inhibitor | 460 (84.2) | 53 (80.3) | 407 (84.8) | 0.498 |
| Betablocker | 501 (91.8) | 57 (86.4) | 444 (92.5) | 0.182 |
| Statin | 525 (96.2) | 53 (80.3) | 472 (98.3) | <0.001 |

ACS: acute coronary syndrome; BMI: body mass index; eGFR: estimated glomerular filtration rate; TNT: Troponin T; MACE: major adverse cardiovascular event; NSTEMI: non-ST elevation myocardial infarction; STEMI: ST elevation myocardial infarction; ACE inhibitors: angiotensin-converting enzyme inhibitor; ARB: angiotensin II receptor blocker; ASA: acetylsalicylic acid.

**Supplementary Table 3.** Associations between baseline CD46 and CD59 and outcomes in patients younger than 75 years at study inclusion

|  |  | MACE  (N=36) | | | HF  (N=13) | | | Total mortality  (N=16) | | |
| --- | --- | --- | --- | --- | --- | --- | --- | --- | --- | --- |
|  | Model | HR | CI | P | HR | CI | P | HR | CI | P |
| CD46 | Model 1 | 1.35 | 1.05 - 1.75 | 0.022 | 1.78 | 1.27 - 2.48 | <0.001 | 1.43 | 0.99 - 2.06 | 0.057 |
|  | Model 2 | 1.36 | 1.04 - 1.77 | 0.023 | 1.79 | 1.26 - 2.54 | 0.001 | 1.44 | 1.00 - 2.09 | 0.052 |
|  | Model 3 | 1.37 | 1.02 - 1.84 | 0.035 | 1.30 | 0.84 - 2.02 | 0.240 | 1.27 | 0.83 - 1.94 | 0.267 |
| CD59 | Model 1 | 1.30 | 1.03 - 1.65 | 0.029 | 1.94 | 1.51 - 2.50 | <0.001 | 1.37 | 0.97 - 1.92 | 0.072 |
|  | Model 2 | 1.28 | 1.00 - 1.64 | 0.051 | 1.90 | 1.47 - 2.47 | <0.001 | 1.33 | 0.93 - 1.90 | 0.114 |
|  | Model 3 | 1.30 | 1.00 - 1.68 | 0.050 | 1.63 | 1.15 – 2.30 | 0.006 | 1.21 | 0.78 - 1.87 | 0.400 |

Model 1: unadjusted

Model 2: age and sex

Model 3: age, sex, CV risk factors (diabetes mellitus, hypertension, smoking), prevalent ACS, prevalent HF, revascularization (PCI/CABG)
